# Self-adaptive iterative method for solving boundedly Lipschitz continuous and strongly monotone variational inequalities

**DOI:** 10.1186/s13660-018-1941-2

**Published:** 2018-12-18

**Authors:** Songnian He, Lili Liu, Aviv Gibali

**Affiliations:** 1 Tianjin Key Laboratory for Advanced Signal Processing, Civil Aviation University of China, Tianjin, China; 2 College of Science, Civil Aviation University of China, Tianjin, China; 3 Department of Mathematics, ORT Braude College, Karmiel, Israel; 4 The Center for Mathematics and Scientific Computation, University of Haifa, Haifa, Israel

**Keywords:** 47J20, 90C25, 90C30, 90C52, Variational inequalities, Self-adaptive iterative methods, Boundedly Lipschitz continuous, Strongly monotone

## Abstract

In this paper we introduce a new self-adaptive iterative algorithm for solving the variational inequalities in real Hilbert spaces, denoted by $\operatorname{VI}(C, F)$. Here $C\subseteq \mathcal{H}$ is a nonempty, closed and convex set and $F: C\rightarrow \mathcal{H}$ is boundedly Lipschitz continuous (i.e., Lipschitz continuous on any bounded subset of *C*) and strongly monotone operator. One of the advantages of our algorithm is that it does not require the knowledge of the Lipschitz constant of *F* on any bounded subset of *C* or the strong monotonicity coefficient a priori. Moreover, the proposed self-adaptive step size rule only adds a small amount of computational effort and hence guarantees fast convergence rate. Strong convergence of the method is proved and a posteriori error estimate of the convergence rate is obtained.

Primary numerical results illustrate the behavior of our proposed scheme and also suggest that the convergence rate of the method is comparable with the classical gradient projection method for solving variational inequalities.

## Introduction

Let $\mathcal{H}$ be a real Hilbert space with inner product $\langle \cdot ,\cdot \rangle $ and induced norm $\Vert \cdot \Vert $, and let *C* be a nonempty, closed and convex subset of $\mathcal{H}$. Let $F:C\rightarrow \mathcal{H}$ be a nonlinear operator. The classical variational inequality problem $\operatorname {VI}(C, F)$ consists of finding a point $x^{*} \in C$ such that

1.1 $$ \bigl\langle Fx^{*},x-x^{*}\bigr\rangle \geq 0, \quad \forall\ x\in C. $$

The variational inequality problem (VIP) was introduced and studied by Fichera [9, 10] (see also [22]). Since then VIPs have been studied and applied in a wide variety of problems arising in different fields, for example, engineering science, structural analysis, economics, optimization, operations research, see [1, 2, 6–11, 14, 16–20, 22, 24–26] and the references therein.

It is easy to verify that, for some $x^{*}\in C$, $x^{*}$ solves the $\operatorname {VI}(C, F)$ if and only if $x^{*}$ satisfies the fixed point equation:

1.2 $$ x^{*}=P_{C}(I-\lambda F)x^{*}, $$

where *I* is the identity operator on $\mathcal{H}$, $P_{C}: \mathcal{H}\rightarrow C$ is the metric projection operator and *λ* is an arbitrary positive constant. Furthermore, if *F* is *η*-strongly monotone and *L*-Lipschitz continuous, i.e., there exist two positive constants *η* and *L* such that

1.3 $$ \langle Fx-Fy, x-y\rangle \geq \eta \Vert x-y \Vert ^{2}, \quad \forall\ x,y\in C, $$

and

1.4 $$ \Vert Fx-Fy \Vert \leq L \Vert x-y \Vert , \quad \forall\ x,y\in C, $$

respectively, then $P_{C}(I-\lambda F): C\rightarrow C$ is a strict contraction with the constant $\sqrt{1-\lambda (2\eta -\lambda L ^{2})}$ for any $\lambda \in (0, \frac{2\eta }{L^{2}})$ (see, for example, Theorem 5 in [4]). Therefore, by (1.2) and Banach’s fixed point theorem, $\operatorname {VI}(C, F)$ has a unique solution and the sequence $\{x_{n}\}_{n=0}^{\infty }$ generated by the gradient projection method (GPM), namely

1.5 $$ x_{0}\in C,\quad\quad x_{n+1}=P_{C}(I- \lambda F)x_{n},\quad n\geq 0, $$

converges strongly to the unique solution of $\operatorname {VI}(C, F)$. The implementation of the gradient projection method (1.5) depends on the “simplicity” of the set *C*, so that the projection operator $P_{C}$ can be easily computed, and on the fact that the strong monotonicity coefficient *η*, the Lipschitz constant *L*, and hence *λ* are all known in advance. In general, this is not the case and therefore many strategies have been developed in the literature to overcome this obstacle; for example, Gibali et al. [13] proposed a relaxed projection method inspired by the work of Fukushima [11]. In away to deal with the second difficulty related to not knowing the parameters, *η*, *L* and *λ*, one can adopt the variable parameter gradient projection method (VPGPM), which approximates *λ* in (1.5) by a sequence $\{\lambda _{n}\}_{n=0}^{\infty }$ satisfying

1.6 $$ \lim_{n\rightarrow \infty }\lambda_{n}=0\quad \text{and} \quad \sum_{n=0}^{\infty } \lambda_{n}=\infty . $$

The above aspects attract much attention and have been studied intensively; for some direct extensions of Fukushima’s method, the readers are refereed to the works of Censor and Gibali [5], Cegielski et al. [3] and Gibali et al. [12]. Related results with Lipschitz continuous and strongly monotone VIPs in real Hilbert spaces, see the relaxed projection methods of He and Yang [19] and He and Tian [17]. For Lipschitz continuous and monotone VIPs in real Hilbert spaces, see [13], which has been extended recently by Cai et al. [2] to Banach spaces.

We point out that most of the algorithms mentioned above use variable parameter sequences satisfying (1.6), this might be essential when the feasible set *C* is more complex and thus the relaxation projection technique has to be used. On the other hand, when *C* is easy to project onto and the constants *η* and *L* are unknown, the usage of the parameter sequence $\{\lambda_{n}\}_{n=0}^{\infty }$ satisfying (1.6) is not a good choice due to the computational effort of doing so. So, our main motivation of this paper is to propose a new simple and fast converging iterative algorithm with self-adaptive parameter selection.

One of the main advantages of our new proposed method is that it does not require a priori the knowledge of the Lipschitz constant of *F* on any bounded subset of *C* or the strong monotonicity coefficient. Moreover, the proposed self-adaptive step size rule only adds a small amount of computational effort and hence guarantees fast convergence rate. Strong convergence of the method is proved and a posteriori error estimate of the convergence rate is obtained. Primary numerical results demonstrate the applicability and efficiency of the algorithm.

As used in our VIP, we present next an example of a nonlinear operator which is strongly monotone and boundedly Lipschitz continuous. Consider the operator $F: C\rightarrow \mathbb{R}^{2}$ defined by

$$ F(x,y)=\bigl(x+y+x^{3}, -x+y+y^{5}\bigr)^{\top }, $$

where $C=\{(x,y)^{\top }\in \mathbb{R}^{2}\mid x, y\geq 0\}$. For any $(x,y)^{\top }, (u,v)^{\top }\in C$, by the mean value theorem, we deduce that

$$\begin{aligned} &\bigl\langle F(x,y)-F(u,v), (x,y)^{\top }-(u,v)^{\top } \bigr\rangle \\ &\quad = \bigl[(x-u)^{2}+(y-v)^{2}\bigr]+\bigl(x ^{3}-u^{3}\bigr) (x-u)+\bigl(y^{5}-v^{5} \bigr) (y-v) \\ &\quad \geq \bigl[(x-u)^{2}+(y-v)^{2}\bigr] \\ &\quad = \bigl\Vert (x,y)^{\top }-(u,v)^{\top } \bigr\Vert ^{2}. \end{aligned}$$

This means that *F* is 1-strongly monotone on *C*. Set $f(x,y)=(x+y, -x+y)^{\top }$, $g(x,y)=(x^{3}, y^{5})^{\top }$, and $B_{\sigma }:=\{(x,y)^{ \top }\mid 0\leq x, y\leq \sigma \}$ for any $\sigma \geq 1$. We have

1.7 $$ \bigl\Vert f(x,y)-f(u,v) \bigr\Vert =\sqrt{2} \bigl\Vert (x,y)^{\top }-(u,v)^{\top } \bigr\Vert , \quad \forall\ (x,y)^{\top }, (u,v)^{\top }\in C. $$

Using again the mean value theorem, we easily obtain

1.8 $$ \begin{aligned}[b] \bigl\Vert g(x,y)-g(u,v) \bigr\Vert &= \sqrt{\bigl(x^{3}-u^{3}\bigr)^{2}+ \bigl(y^{5}-v^{5}\bigr)^{2}} \\ &\leq 5\sigma^{4} \bigl\Vert (x,y)^{\top }-(u,v)^{\top } \bigr\Vert , \quad \forall\ (x,y)^{\top }, (u,v)^{\top }\in B_{\sigma }. \end{aligned} $$

Combining (1.7) and (1.8) leads to

$$ \bigl\Vert F(x,y)-F(u,v) \bigr\Vert \leq \bigl(\sqrt{2}+5 \sigma^{4}\bigr) \bigl\Vert (x,y)^{\top }-(u,v)^{ \top } \bigr\Vert , \quad \forall\ (x,y)^{\top }, (u,v)^{\top }\in B_{\sigma }, $$

which implies that *F* is boundedly Lipschitz continuous on *C*. However, *F* is not Lipschitz continuous on *C*. Indeed, it is very easy to see that

$$ \frac{ \Vert F(x,0)-F(0,0) \Vert }{ \Vert (x,0)^{\top }-(0,0)^{\top } \Vert }\geq \frac{x ^{3}-\sqrt{2}x}{x}=x^{2}-\sqrt{2}\rightarrow + \infty , $$

as $x\rightarrow +\infty $.

The outline of the paper is as follows. In Sect. 2, we recall some basic definitions and results which are useful for our analysis. Our self-adaptive iterative algorithm is presented and analyzed in Sect. 3. Then, in Sect. 4, three numerical experiments which demonstrate and compare our algorithm’s performance with two related methods are presented. Final conclusions are given in Sect. 5.

## Preliminaries

In this section, we list some concepts and tools that will be used in the proofs of our main results. In the rest of this paper, we always denote by $\mathcal{H}$ a real Hilbert space and denote by *I* the identity operator on $\mathcal{H}$. Also, we will use the following notations:

(i) → denotes strong convergence.
(ii) ⇀ denotes weak convergence.
(iii) $\omega_{w}(x_{n}) =\{x\mid \exists\ \{x_{n_{k}}\}_{k=1} ^{\infty }\subset \{x_{n}\}_{n=1}^{\infty }\text{ such that } x_{n_{k}} \rightharpoonup x\}$ denotes the weak *ω*-*limit* set of $\{x_{n}\}_{n=1}^{\infty }$.
(iv) $S(x,r)$ denotes the closed ball with center $x\in \mathcal{H}$ and radius $r>0$.

Let *C* be a nonempty closed convex subset of a real Hilbert space $\mathcal{H}$. Then, for any $x\in \mathcal{H}$, there is a unique point $z\in C$ such that $\Vert z-x\Vert \leq \Vert y-x\Vert $ for all $y\in C$, this vector *z*, denoted by $P_{C}x$, is called the metric projection of *x* onto *C* and the operator $P_{C}: \mathcal{H}\rightarrow C$ is called the metric projection operator onto *C*. It is well known that the projection operator $P_{C}$ is non-expansive; namely,

$$ \Vert P_{C}x-P_{C}y \Vert \leq \Vert x-y \Vert , \quad \forall\ x,y\in \mathcal{H}. $$

In fact, $P_{C}$ is also a firmly nonexpansive mapping, i.e.,

2.1 $$ \Vert P_{C}x-P_{C}y \Vert ^{2}\leq \Vert x-y \Vert ^{2}- \bigl\Vert (x-P_{C}x)-(y-P_{C}y) \bigr\Vert ^{2}, \quad \forall\ x,y\in \mathcal{H}. $$

It is well known that $P_{C}x$ is characterized [15, Sect. 3] by the inequality (for $x\in H$)

2.2 $$ \langle x-P_{C}x,y-P_{C}x\rangle \leq 0, \quad \forall\ y\in C. $$

### Lemma 2.1

*The following inequality holds*:

2.3 $$ \Vert x+y \Vert ^{2}\leq \Vert x \Vert ^{2}+2\langle y, x+y\rangle , \quad \forall\ x,y\in \mathcal{H}. $$

### Lemma 2.2

([21])

*Let* $T: C\rightarrow C$ *be a nonexpansive mapping*. *Then* $I-T$ *is demiclosed at* 0 *in the sense that if* $\{x_{n}\}_{n=1}^{\infty }$ *is a sequence in* *C* *such that* $x_{n}\rightharpoonup x$ *and* $\Vert x_{n}-Tx _{n}\Vert \rightarrow 0$ *as* $n\rightarrow \infty $, *it follows that* $x-Tx=0$, *i*.*e*., $x\in \operatorname {Fix}(T)$. *Here* $\operatorname {Fix}(T)=\{x\in \mathcal{H}\mid Tx=x \}$ *is the set of fixed points of T*.

### Definition 2.3

A mapping $F: C\rightarrow \mathcal{H}$ is said to be boundedly Lipschitz continuous, if *F* is Lipschitz continuous on any bounded subset *B* of *C*, i.e., there exists some $L_{B}>0$ ($L_{B}$ is relevant with subset *B*) such that

2.4 $$ \Vert Fx-Fy \Vert \leq L_{B} \Vert x-y \Vert , \quad \forall\ x,y\in B. $$

### Lemma 2.4

([23])

*Assume* $\{a_{n}\}_{n=0}^{\infty }$ *is a sequence of nonnegative real numbers such that*

2.5 $$\begin{aligned} a_{n+1} \leq (1-\gamma_{n})a_{n} + \gamma_{n}\sigma_{n}, \quad n\geq 0, \end{aligned}$$

*where* $\{\gamma_{n}\}_{n=0}^{\infty }$ *is a sequence in* $(0,1)$ *and* $\{\sigma_{n}\}_{n=0}^{\infty }$ *is a sequence of real numbers such that*

(i) $\sum_{n=0}^{\infty }\gamma_{n} = \infty $,
(ii) $\sum_{n=1}^{\infty }\vert \gamma_{n}\sigma_{n}\vert = \infty $, *or* $\limsup_{n\rightarrow \infty }\sigma_{n}\leq 0$.

*Then* $\lim_{n\rightarrow \infty }a_{n}=0$.

### Theorem 2.5

([18])

*Let* *C* *be a nonempty closed convex subset of a real Hilbert space* $\mathcal{H}$. *If* $F: C\rightarrow \mathcal{H}$ *is a strongly monotone and boundedly Lipschitz continuous operator*, *then the variational inequality* $\operatorname {VI}(C, F)$ *has a unique solution*.

## The self-adaptive iterative algorithm

Let $\mathcal{H}$ be a real Hilbert space with inner product $\langle \cdot ,\cdot \rangle $ and induced norm $\Vert \cdot \Vert $, and let *C* be a nonempty closed convex subset of $\mathcal{H}$. Let $F: C\rightarrow \mathcal{H}$ be a strongly monotone and boundedly Lipschitz continuous operator. Throughout this section, we always assume that we do not need to know or to estimate its strong monotonicity coefficient *η* and the Lipschitz constant $L_{B}$ on any bounded subset *B* of *C*. Also, we always assume that the projection operator $P_{C}$ is easy to calculate. Using Theorem 2.5, $\operatorname {VI}(C, F)$ has a unique solution, denoted by $x^{*}$.

Now we are ready to present our self-adaptive iterative algorithm for solving $\operatorname {VI}(C, F)$.

### Algorithm 3.1

(Self-adaptive iterative algorithm)

1. Choose $x_{0}\in C$ arbitrarily and set $n:=1$. Calculate $x_{1}$ by
  $$ x_{1}=P_{C}\bigl(x_{0}-F(x_{0}) \bigr). $$
  If $x_{1}=x_{0}$, then $x_{0}$ is the unique solution of $\operatorname {VI}(C, F)$ and stop the algorithm.
  Otherwise, set
  $$ \eta_{0}=\frac{\langle F(x_{1})-F(x_{0}),x_{1}-x_{0}\rangle }{ \Vert x_{1}-x _{0} \Vert ^{2}},\quad\quad L_{0}= \frac{ \Vert F(x_{1})-F(x_{0}) \Vert }{ \Vert x_{1}-x_{0} \Vert }, \quad \text{and} \quad \mu_{0}=\frac{\eta_{0}}{L_{0}^{2}}. $$
2. Given the current iterate $x_{n}$, compute
  $$\begin{aligned}& \eta_{n}= \textstyle\begin{cases} \min \{\eta_{n-1}, \frac{\langle F(x_{n})-F(x_{n-1}),x_{n}-x_{n-1} \rangle }{ \Vert x_{n}-x_{n-1} \Vert ^{2}}, \frac{\langle F(x_{n})-F(x_{0}),x _{n}-x_{0}\rangle }{ \Vert x_{n}-x_{0} \Vert ^{2}}\} , &\text{if } x_{n}\neq x_{0}, \\ \min \{\eta_{n-1}, \frac{\langle F(x_{n})-F(x_{n-1}),x_{n}-x_{n-1} \rangle }{ \Vert x_{n}-x_{n-1} \Vert ^{2}}\}, & \text{if } x_{n}= x_{0}, \end{cases}\displaystyle \\& L_{n}= \textstyle\begin{cases} \max \{L_{n-1}, \frac{ \Vert F(x_{n})-F(x_{n-1}) \Vert }{ \Vert x_{n}-x_{n-1} \Vert }, \frac{ \Vert F(x_{n})-F(x_{0}) \Vert }{ \Vert x_{n}-x_{0} \Vert }\} ,& \text{if } x_{n}\neq x_{0}, \\ \max \{L_{n-1}, \frac{ \Vert F(x_{n})-F(x_{n-1}) \Vert }{ \Vert x_{n}-x_{n-1} \Vert }\}, & \text{if } x_{n}= x_{0}, \end{cases}\displaystyle \end{aligned}$$
  and
  $$ \mu_{n}=\frac{\eta_{n}}{L_{n}^{2}}. $$
3. Update the next iterate as
  3.1 $$ x_{n+1}=P_{C}\bigl(x_{n}- \mu_{n}F(x_{n})\bigr),\quad n\geq 1. $$
  If $x_{n+1}=x_{n}$, STOP, $x_{n}$ is the unique solution of $\operatorname {VI}(C, F)$.
  Otherwise, set $n:=n+1$ and return to Step 2.

### Remark 3.2

We make the following observations for Algorithm 3.1.

1. It is easy to see by a simple induction that the sequences $\{\eta_{n}\}_{n=0}^{\infty }$, $\{L_{n}\}_{n=0}^{\infty }$, and $\{\mu_{n}\}_{n=0}^{\infty }$ are well defined. Also the calculations of $\eta_{n}$, $L_{n}$, and $\mu_{n}$ only add a small amount of computational load. Indeed, for any $n\geq 1$, the values of $\{F(x_{k})\}_{k=0}^{n}$ have been obtained in the previous calculations.
2. Let *η* be the strong monotonicity coefficient of *F*. Then the following properties directly follow from the definitions of $\eta_{n}$, $L_{n}$ and $\mu_{n}$:
  (i) $\{\eta_{n}\}_{n=0}^{\infty }$ is monotone nonincreasing and $\eta_{n}\geq \eta $ for all $n\geq 0$.
  (ii) $\{L_{n}\}_{n=0}^{\infty }$ is monotone nondecreasing and $L_{n}\geq \eta_{n}$ holds for all $n\geq 0$. Particularly, if *F* is *L*-Lipschitz continuous, then $L_{n}\leq L$ holds for all $n\geq 0$.
  (iii) $\{\mu_{n}\}_{n=0}^{\infty }$ is monotone nonincreasing and $\mu_{n}=\frac{\eta_{n}}{L_{n}^{2}}\leq \frac{1}{\eta_{n}}\leq \frac{1}{ \eta }$ holds for all $n\geq 0$. In particular, if *F* is *L*-Lipschitz continuous, then $\mu_{n}\geq \frac{\eta }{L^{2}}$ holds for all $n\geq 0$.

Next we present a strong convergence theorem of Algorithm 3.1.

### Theorem 3.3

*Assume that* *F* *is boundedly Lipschitz continuous and strongly monotone on the feasible set*, *then any sequence* $\{x_{n}\}_{n=0}^{\infty }$ *generated by Algorithm *3.1 *converges strongly to the unique solution* $x^{*}$ *of problem* $\operatorname {VI}(C,F)$.

### Proof

First, we verify that $\{x_{n}\}_{n=0}^{\infty }$ is bounded. For any $n\geq 1$, put $y_{n}=P_{C}(x_{0}-\mu_{n}F(x_{0}))$ and recall the definitions of $\eta_{n}$, $L_{n}$ and $\mu_{n}$. We have from (3.1) that

$$\begin{aligned} \Vert x_{n+1}-y_{n} \Vert ^{2}&= \bigl\Vert P_{C}\bigl(x_{n}-\mu_{n}F(x_{n}) \bigr)-P_{C}\bigl(x_{0}-\mu _{n}F(x_{0}) \bigr) \bigr\Vert ^{2} \\ &\leq \bigl\Vert (x_{n}-x_{0})-\mu_{n} \bigl(F(x_{n})-F(x_{0})\bigr) \bigr\Vert ^{2} \\ &= \Vert x_{n}-x_{0} \Vert ^{2}-2 \mu_{n}\bigl\langle F(x_{n})-F(x_{0}),x _{n}-x_{0} \bigr\rangle +\mu_{n}^{2} \bigl\Vert F(x_{n})-F(x_{0}) \bigr\Vert \\ &\leq \Vert x_{n}-x _{0} \Vert ^{2}-2 \mu_{n}\eta_{n} \Vert x_{n}-x_{0} \Vert ^{2}+\mu_{n}^{2}L_{n}^{2} \Vert x _{n}-x_{0} \Vert ^{2} \\ &= \biggl( 1-\frac{\eta_{n}^{2}}{L_{n}^{2}} \biggr) \Vert x _{n}-x_{0} \Vert ^{2} \\ &\leq \biggl( 1-\frac{1}{2}\frac{\eta_{n}^{2}}{L_{n} ^{2}} \biggr) ^{2} \Vert x_{n}-x_{0} \Vert ^{2}. \end{aligned}$$

Hence

3.2 $$ \Vert x_{n+1}-y_{n} \Vert \leq \biggl( 1- \frac{1}{2}\frac{\eta_{n}^{2}}{L_{n} ^{2}} \biggr) \Vert x_{n}-x_{0} \Vert . $$

Also, we have

$$\begin{aligned} \Vert x_{n+1}-x_{0} \Vert &\leq \Vert x_{n+1}-y_{n} \Vert + \Vert y_{n}-x_{0} \Vert \\ &\leq \biggl( 1- \frac{1}{2}\frac{\eta_{n}^{2}}{L_{n}^{2}} \biggr) \Vert x_{n}-x_{0} \Vert + \bigl\Vert P _{C} \bigl(x_{0}-\mu_{n}F(x_{0})\bigr)-P_{C}x_{0} \bigr\Vert \\ &\leq \biggl( 1-\frac{1}{2}\frac{ \eta_{n}^{2}}{L_{n}^{2}} \biggr) \Vert x_{n}-x_{0} \Vert +\mu_{n} \bigl\Vert F(x_{0}) \bigr\Vert \\ &= \biggl( 1-\frac{1}{2}\frac{\eta_{n}^{2}}{L_{n}^{2}} \biggr) \Vert x_{n}-x_{0} \Vert +\frac{\eta_{n}^{2}}{2L_{n}^{2}}\frac{2}{\eta_{n}} \bigl\Vert F(x_{0}) \bigr\Vert \\ & \leq \max \biggl\{ \Vert x_{n}-x_{0} \Vert , \frac{2}{\eta_{n}} \bigl\Vert F(x_{0}) \bigr\Vert \biggr\} \\ &\leq \max \biggl\{ \Vert x_{n}-x_{0} \Vert , \frac{2}{\eta } \bigl\Vert F(x_{0}) \bigr\Vert \biggr\} . \end{aligned}$$

By induction, we obtain

$$ \Vert x_{n+1}-x_{0} \Vert \leq \max \biggl\{ \Vert x_{1}-x_{0} \Vert ,\frac{2}{\eta } \bigl\Vert F(x _{0}) \bigr\Vert \biggr\} ,\quad \forall\ n\geq 1, $$

which means that $\{x_{n}\}_{n=0}^{\infty }$ is bounded. So is $\{F(x_{n})\}_{n=0}^{\infty }$ due to the fact that *F* is boundedly Lipschitz continuous.

Second, we show that $\{x_{n}\}_{n=0}^{\infty }$ is a Cauchy sequence. In fact, for any $n\geq 2$, we have from (3.1) that

$$\begin{aligned} \Vert x_{n+1}-x_{n} \Vert &= \bigl\Vert P_{C}\bigl(x_{n}-\mu_{n}F(x_{n}) \bigr)-P_{C}\bigl(x_{n-1}-\mu _{n-1}F(x_{n-1}) \bigr) \bigr\Vert \\ &\leq \bigl\Vert (x_{n}-x_{n-1})-\mu_{n} \bigl(F(x_{n})-F(x _{n-1})\bigr)+(\mu_{n-1}- \mu_{n})F(x_{n-1}) \bigr\Vert \\ &\leq \bigl\Vert (x_{n}-x_{n-1})-\mu _{n} \bigl(F(x_{n})-F(x_{n-1})\bigr) \bigr\Vert +( \mu_{n-1}-\mu_{n}) \bigl\Vert F(x_{n-1}) \bigr\Vert . \end{aligned}$$

Noting the definitions of $\eta_{n}$, $L_{n}$ and $\mu_{n}$ again, an argument very similar to getting (3.2) yields

$$ \bigl\Vert (x_{n}-x_{n-1})-\mu_{n} \bigl(F(x_{n})-F(x_{n-1})\bigr) \bigr\Vert \leq \biggl( 1- \frac{1}{2}\frac{\eta_{n}^{2}}{L_{n}^{2}} \biggr) \Vert x_{n}-x_{n-1} \Vert . $$

Consequently,

3.3 $$ \Vert x_{n+1}-x_{n} \Vert \leq \biggl( 1- \frac{1}{2}\frac{\eta_{n}^{2}}{L_{n} ^{2}} \biggr) \Vert x_{n}-x_{n-1} \Vert +(\mu_{n-1}-\mu_{n}) \bigl\Vert F(x_{n-1}) \bigr\Vert . $$

We denote by *B* the closed convex hull of the sequence $\{x_{n}\} _{n=0}^{\infty }$ and by $L_{B}$ the Lipschitz constant of *F* restricted to *B*, respectively. Noting $\eta_{n}\geq \eta $ and $L_{n}\leq L_{B}$ ($n\geq 0$), we obtain from (3.3) that

3.4 $$ \Vert x_{n+1}-x_{n} \Vert \leq \biggl( 1- \frac{1}{2}\frac{\eta^{2}}{L_{B}^{2}} \biggr) \Vert x_{n}-x_{n-1} \Vert +(\mu_{n-1}-\mu_{n})M, $$

where $M=\sup \{\Vert F(x_{n})\Vert \}_{n=0}^{\infty }<+\infty $. On the other hand, $\sum_{n=1}^{\infty }\vert \mu_{n-1}-\mu_{n}\vert <+\infty $ holds since $\{\mu_{n}\}_{n=0}^{\infty }$ is monotone nonincreasing. Using Lemma 2.4, it follows that $\Vert x_{n+1}-x_{n}\Vert \rightarrow 0$ as $n\rightarrow \infty $. For any integers *n* and *m* such that $m>n\geq 2$, it follows from (3.4) that

$$ \frac{\eta^{2}}{2L_{B}^{2}}\sum_{k=n}^{m-1} \Vert x_{k}-x_{k+1} \Vert \leq \biggl( 1-\frac{\eta^{2}}{2L_{B}^{2}} \biggr) \Vert x_{n-1}-x_{n} \Vert +(\mu_{n-1}- \mu_{m-1})M. $$

Furthermore, we get

3.5 $$ \Vert x_{n}-x_{m} \Vert \leq \frac{2L_{B}^{2}}{\eta^{2}} \biggl\{ \biggl( 1-\frac{ \eta^{2}}{2L_{B}^{2}} \biggr) \Vert x_{n-1}-x_{n} \Vert +(\mu_{n-1}- \mu_{m-1})M \biggr\} . $$

From (3.5), it is easy to see that $\{x_{n}\}_{n=0}^{\infty }$ is a Cauchy sequence due to the fact that $\Vert x_{n+1}-x_{n}\Vert \rightarrow 0$ and $\lim_{n\rightarrow \infty }\mu_{n}$, denoted by $\mu^{*}$, exists.

Finally, we prove $x_{n}\rightarrow x^{*}$ ($n\rightarrow \infty $). Set $\lim_{n\rightarrow \infty }x_{n}=z$. Using the relations

$$ \frac{\eta }{L_{B}^{2}}\leq \mu_{n}=\frac{\eta_{n}}{L_{n}^{2}}\leq \frac{1}{ \eta },\quad \forall\ n\geq 0, $$

we assert that $\mu^{*}=\lim_{n\rightarrow \infty }\mu_{n}\geq \frac{ \eta }{L_{B}^{2}}$. Taking $n\rightarrow \infty $ in (3.1), we obtain

$$ z=P_{C}\bigl(z-\mu^{*}F(z)\bigr). $$

This implies that $z\in C$ is a solution of $\operatorname {VI}(C, F)$. Using the uniqueness of the solution of the $\operatorname {VI}(C, F)$, we assert that $z=x^{*}$, and this completes the proof. □

To present a complete convergence analysis of Algorithm 3.1, the next theorem establishes the algorithm’s convergence rate.

### Theorem 3.4

*Assume that* *F* *is boundedly Lipschitz continuous and strongly monotone on the feasible set and the sequence* $\{x_{n}\}_{n=0}^{\infty }$ *is generated by Algorithm *3.1. *Then the following a posteriori error estimate holds*:

3.6 $$ \bigl\Vert x_{n}-x^{*} \bigr\Vert \leq \frac{2L_{B}^{2}}{\eta^{2}} \biggl\{ \biggl( 1-\frac{ \eta^{2}}{2L_{B}^{2}} \biggr) \Vert x_{n-1}-x_{n} \Vert +\bigl(\mu_{n-1}- \mu^{*}\bigr)M \biggr\} , \quad \forall\ n\geq 2, $$

*where* *η* *is the strong monotonicity coefficient of* *F*, *and the constants* $L_{B}$, $\mu^{*}$ *and* *M* *are the same as above*.

### Proof

Observe that this estimate can be easily obtained by letting $m\rightarrow \infty $ in (3.5). □

Since a Lipschitz continuous operator is obviously boundedly Lipschitz continuous, the following results are straightforward.

### Corollary 3.5

*Assume that* *F* *is Lipschitz continuous and strongly monotone on the feasible set*, *then the sequence* $\{x_{n}\}_{n=0}^{\infty }$ *generated by Algorithm *3.1 *converges strongly to the unique solution* $x^{*}$ *of problem* $\operatorname {VI}(C,F)$.

### Corollary 3.6

*Assume that* *F* *is Lipschitz continuous and strongly monotone on the feasible set and the sequence* $\{x_{n}\}_{n=0}^{\infty }$ *is generated by Algorithm *3.1. *Then the following a posteriori error estimate holds*:

3.7 $$ \bigl\Vert x_{n}-x^{*} \bigr\Vert \leq \frac{2L^{2}}{\eta^{2}} \biggl\{ \biggl( 1-\frac{\eta ^{2}}{2L^{2}} \biggr) \Vert x_{n-1}-x_{n} \Vert +\bigl(\mu_{n-1}- \mu^{*}\bigr)M \biggr\} , \quad \forall\ n\geq 2, $$

*where the constants* $\mu^{*}$ *and* *M* *are the same as above*, *and* *L* *and* *η* *is the Lipschitz constant and strong monotonicity coefficient of* *F*, *respectively*.

## Numerical results

In this section, we present three numerical examples which demonstrate the performance of the self-adaptive iterative algorithm (Algorithm 3.1). All implementations and testing are preformed with Matlab R2014b on an HP Pavilion notebook with Intel(R) Core(TM) i5-3230M CPU@2.60 GHz and 4 GB RAM running on Windows 10 Home Premium operating system.

### Example 1

Consider the variational inequality problem $\operatorname {VI}(C, F)$ (1.1) with the set $C :=\{(x,y)\mid x^{2}+y^{2}\leq 1\}$ and $F:C\rightarrow R^{2}$ defined by $F(x,y)=(2x+2y+\sin (x),-2x+2y+\sin (y))^{ \top }$, $\forall\ (x,y)^{\top }\in C$.

One can easily verify that *F* is strongly monotone and Lipschitz continuous with strong monotonicity coefficient $\eta =1$ and Lipschitz constant $L=1+\sqrt{8}$, respectively, and $\operatorname {VI}(C, F)$ has the unique solution $x^{*}=(0,0)^{\top }$. Now we compare the numerical performance of Algorithm 3.1, GPM (with the known constant $\lambda =\frac{ \eta }{L^{2}}$) and VPGPM (with the variable parameter sequence $\{\lambda_{n}\}_{n=0}^{\infty }=\{\frac{1}{n+1}\}_{n=0}^{ \infty }$). Since the exact solution of the $\operatorname {VI}(C,F)$ is known, we naturally use

4.1 $$ E_{n}:= \bigl\Vert x_{n}-x^{*} \bigr\Vert $$

to measure the error of the *n*th iterate $x_{n}$. The numerical results of Algorithm 3.1, GPM and VPGPM with the same initial guess $x_{0}=(1, 0)^{\top }$ for solving Example 1 are listed in Table 1, where “Iter.” denotes the number of iterations.

**Table 1:** Comparison of Algorithm 3.1 with GPM and VPGPM

$E_{n}$	Iter.			CPU (in s)		
	VPGPM	GPM	Algorithm 3.1	VPGPM	GPM	Algorithm 3.1
1⋅10^−1^	7	11	5	0.000064	0.000488	0.001099
1⋅10^−2^	15	22	9	0.000126	0.000554	0.001709
1⋅10^−3^	31	33	13	0.000213	0.000621	0.002412
1⋅10^−4^	66	44	17	0.000420	0.000684	0.003223
1⋅10^−5^	141	54	21	0.000855	0.000746	0.003691
1⋅10^−6^	302	65	25	0.001784	0.000820	0.004325
1⋅10^−7^	649	76	29	0.003787	0.000900	0.004826
1⋅10^−8^	1398	87	33	0.008293	0.001003	0.005116

**Figure 1:**
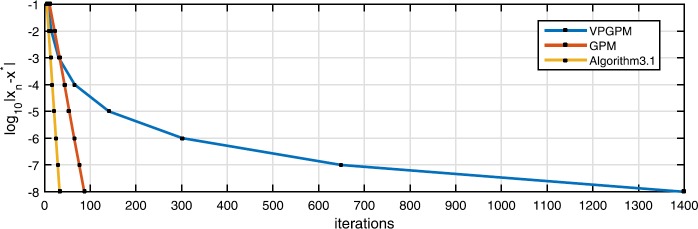
Illustrations and comparison of three algorithms for Example 1

Next, in Fig. 1 we graphically present the numerical performance of the above three algorithms.

From Table 1 and Fig. 1, we conclude that the VPGPM performs the worst, regardless of the number of iterations or the computing time, and Algorithm 3.1 and the GPM are roughly the same since Algorithm 3.1 needs the least number of iterations, while the GPM needs the shortest computing time. Although Algorithm 3.1 requires a little longer computing time than GPM due to parameter self-adaptive selection, Algorithm 3.1 still shows obvious superiority, not only because it requires the least number of iterations, but also because it does not need to know the constants *L* and *η*.

### Example 2

Consider the variational inequality problem $\operatorname {VI}(C, F)$ (1.1) with the set $C :=\{(x,y)\mid x\geq 0,y\geq 0\}$ and $F:C\rightarrow R^{2}$ defined by $F(x,y)=(2x+2y+\exp (x),-2x+2y+\exp (y))^{\top }$, $\forall\ (x,y)^{\top }\in C$.

It is easy to see that *F* is strongly monotone and boundedly Lipschitz continuous on *C* and $x^{*}=(0,0)^{\top }$ is the unique solution. Since *F* is not Lipschitz continuous on *C*, so GPM and VPGPM are not applicable for this example. Choosing the starting point $x_{0}=(1,1)^{ \top }$ and using Algorithm 3.1 to solve this example, we find that the exact solution $x^{*}=(0,0)^{\top }$ can be obtained by only one iteration.

### Example 3

Consider the variational inequality problem $\operatorname {VI}(C, F)$ (1.1) with the set $C :=\{(x,y)\mid x\geq 0\}$ and $F:C\rightarrow R^{2}$ defined by $F(x,y)=(2x+2y+\exp (x),-2x+2y+\exp (y))^{\top }$, $\forall\ (x,y)^{\top }\in C$.

Similar to Example 2, *F* is also strongly monotone and boundedly Lipschitz continuous on *C*, and GPM and VPGPM are not applicable for this example. On the other hand, we define

4.2 $$ E_{n}:= \Vert x_{n}-x_{n-1} \Vert $$

for this example to measure the error of the *n*th iterate $x_{n}$ since the exact solution of this $\operatorname {VI}(C,F)$ problem is unknown. The numerical results generated by implementing Algorithm 3.1 with the initial guess $x_{0}=(2,1)^{\top }$ for solving Example 3 are listed in Table 2, where “Iter.” also denotes the number of iterations.

**Table 2:** Numerical results of Algorithm 3.1

$E_{n}$	Iter.	CPU (in s)
1⋅10^−1^	3	0.000094
1⋅10^−2^	11	0.000282
1⋅10^−3^	19	0.000479
1⋅10^−4^	28	0.000686
1⋅10^−5^	36	0.000867
1⋅10^−6^	45	0.001082
1⋅10^−7^	54	0.001207
1⋅10^−8^	62	0.001425

The numerical results in Tables 1 and 2 show that the convergence rate of Algorithm 3.1 for solving boundedly Lipschitz continuous variational inequalities is almost the same as that of GPM for solving Lipschitz continuous variational inequalities.

## Conclusions

In this paper, in the setting of Hilbert spaces, a new self-adaptive iterative algorithm is proposed for solving $\operatorname {VI}(C, F)$ governed by boundedly Lipschitz continuous and strongly monotone operator $F: C\rightarrow \mathcal{H}$ under the assumption that $P_{C}$ has a closed-form formula. The advantages of our algorithm are not only having no need to know or estimate the strong monotonicity coefficient and Lipschitz constant on any bounded subset of the feasible set, but also having a fast convergence rate because the parameter self-adaptive selection process only adds a small amount of computational effort. Currently, as far as we know, such algorithms for solving strongly monotone and boundedly Lipschitz continuous variational inequalities have not been considered before.
